# Role of Endoscopic Ultrasound-Guided Radiofrequency Ablation in Pancreatic Lesions: Where Are We Now and What Does the Future Hold?

**DOI:** 10.3390/cancers16213662

**Published:** 2024-10-30

**Authors:** Radhika Chavan, Nirav Thosani, Shivangi Kothari

**Affiliations:** 1 Ansh Clinic, Ahmedabad 380008, India; drradhikachavan@gmail.com; 2 The University of Texas Health Science Center, 7000 Fannin, Suite 1706, Houston, TX 77030, USA; nirav.thosani@uth.tmc.edu; 3 University of Rochester Medical Center, 601 Elmwood Avenue, Rochester, NY 14642, USA

**Keywords:** endoscopic ultrasound, radiofrequency ablation

## Abstract

Endoscopic ultrasound-guided radiofrequency ablation (EUS-RFA) has emerged as an effective and minimally invasive treatment for pancreatic lesions, particularly in patients at high surgical risk. Utilizing thermal energy, RFA induces the coagulative necrosis of the tissue and potentially triggers immunomodulation by releasing intracellular antigens. Numerous studies have confirmed the technical feasibility, safety, and efficacy of EUS-RFA in pancreatic neuroendocrine tumors and premalignant cystic lesions, with an acceptable profile of adverse events. The technique’s potential immunomodulatory effects offer intriguing implications for the treatment of advanced pancreatic malignancies, encouraging further evaluation. This review paper aims to highlight the EUS-RFA principles, technology, and clinical applications in various pancreatic lesions and safety, and the future research directions.

## Simple Summary

Pancreatic tumors are increasingly being detected due to widespread use of imaging in healthcare. Pancreatic tumor can be benign, pre-malignant, and malignant. Surgery is the mainstay for malignant and premalignant pancreatic tumors. However, it is invasive and associated with numerous complications. Radiofrequency ablation uses high thermal energy to induce coagulative necrosis of the tissue. Endoscopic ultrasound-guided radiofrequency ablation (EUS-RFA) is a minimally invasive technique that can serve as an alternative to surgery, particularly for patients at high surgical risk or those who refuse surgery. This review article explores the utility of EUS-RFA in the treatment of pancreatic tumors, such as nonfunctional neuroendocrine tumor, insulinoma (up to 2 cm), and premalignant cystic lesion. It also discusses several aspects of EUS-RFA and future perspectives that could be valuable in clinical practice, thereby improving patient outcomes.

## 1. Introduction

**Figure 1:**
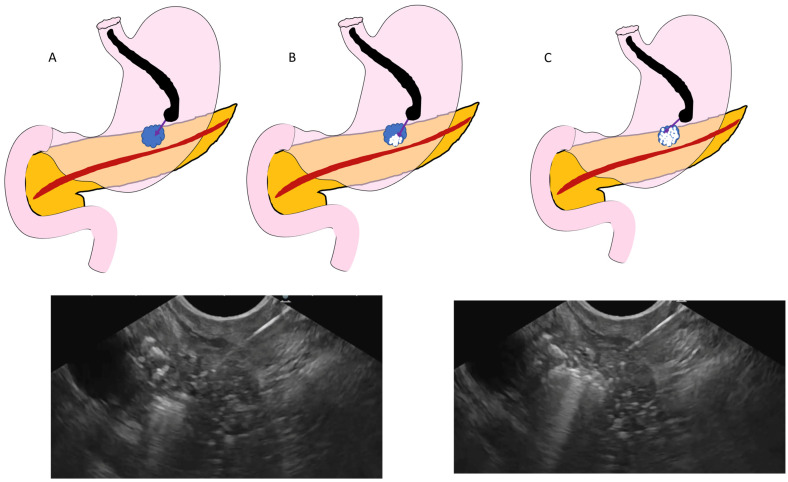
Ablation of pancreas neoplasia with EUS RFA. Appearance of hyperechoic bubbles seen on application of EUS-RFA. The most distal part of tumor is targeted first. Following that, the needle is withdrawn and the proximal part is targeted. (**A**) Illustration showing a pancreatic tumor is targeted with an RFA needle using a linear echoendoscope. (**B**) Upon activation of RFA energy, hyperechoic bubbles are seen within tumor, as seen in the corresponding EUS image. (**C**) Ablation is performed using the fanning technique. Illustration shows hyperechoic bubbles covering most of the tumor, as seen in the corresponding EUS image.

The detection of pancreatic neoplasia has increased significantly due to more widespread use of cross sectional imaging in clinical and healthcare screening [1]. Active surveillance or surgery is typically recommended, based on the tumor size and its malignant potential [2,3]. Although surgery remains the definitive treatment for pancreatic tumors, it is invasive and associated with significant morbidity and mortality [4,5]. Though pancreatic parenchyma preserving surgery like enucleation and limited resection techniques are available for small and benign tumors, only a few patients are candidates for such surgeries. Endoscopic ultrasound (EUS) guided ablation of pancreatic lesions offers a minimally invasive and safe alternative that potentially balances the risk between the “undertreatment” associated with active surveillance and the “overtreatment” involved in surgical intervention for small pancreatic lesion. By enabling direct visualization and accurate targeting, EUS-guided ablation minimizes the risk to surrounding tissues and maximizes the therapeutic efficacy (Figure 1). These ablation techniques include injective ablation (using ethanol or other ablative agents), radiofrequency ablation, photodynamic therapy, and laser ablation. Among these, EUS-guided radiofrequency ablation (EUS-RFA) is favored for its superior safety profile and promising outcomes [6,7].

EUS-RFA in the normal pancreas was initially described by Goldberg and colleagues in 1999, in 13 anesthetized Yorkshire pigs [8]. RFA current (285 ± 120 mA) was delivered with a 19-gauge needle for 6 min, and coagulative necrosis was documented on the necropsy. Since this foundational study, numerous investigations have been conducted on various pancreatic lesions including pancreatic neuroendocrine tumor (PNET), pancreatic cystic lesion (PCL), adenocarcinoma, and pancreatic metastasis, employing a range of RFA devices [6,9,10,11,12,13,14]. EUS-RFA’s safety profile and immediate symptomatic improvement in insulinoma have paved the way for the continuous research of EUS-RFA in PNET [15]. In addition to the coagulative necrosis of the tissue, RFA is believed to possess immunomodulatory properties, making it a subject of interest for treating advanced pancreatic malignancies typically resistant to chemo-radiotherapy [16,17,18,19,20]. This technique has been progressively refined since its inception, demonstrating a favorable safety profile and efficacy across various studies. In this article, we will review important studies on EUS-RFA and discuss the RFA devices and techniques, the clinical outcome of EUS-RFA in various pancreatic lesions, and the future directions of RFA.

## 2. Principles of EUS-RFA

The pancreas is an extremely thermosensitive organ and is located in the vicinity of major blood vessels and vital organs. The mechanism of RFA involves the application of thermal energy using high frequency alternating current to induce targeted destruction of abnormal pancreatic tissue, while minimizing damage to the surrounding organ. This process is facilitated by the use of dedicated RFA probe under EUS guidance. The core principles underpinning RFA in the pancreatic lesion include:

1. Thermal coagulative necrosis: The RFA platform uses high frequency (350–500 kHz) alternating current to generate heat of 60–100 °C within a targeted tissue to cause coagulative necrosis [21,22]. It causes irreversible cell death leading to apoptosis.
2. Thermal Diffusivity Effect: The effectiveness of RFA relies on the “thermal diffusivity effect” which is governed by thermal conductibility of the lesion being treated. The dispersion of energy into the surrounding pancreatic parenchyma is prevented by the lesion, thus preventing the thermal damage to normal parenchyma or surrounding structures [23,24].
3. Zonal phenomenon: The application of RFA results in the creation of three distinct zones within and around the targeted lesion [25,26,27].
  - A central zone of coagulative necrosis directly around the needle point, where tissue death occurs due to intense heat.
  - A middle zone of parenchymal hypochromia or transitional zone indicating less severe thermal effects. Over a period, this transitional zone becomes a fibrotic ring separating normal pancreatic parenchyma and necrotic tissue [17].
  - An outermost unaffected zone, where pancreatic tissue remains intact and unharmed.
4. Increasing energy deposition and minimization of charring: The extent of the ablation zone depends on both the ablation power and duration of the ablation. Experimental studies have shown that the size of ablated area is inversely proportional to the power applied, with maximal coagulative necrosis achieved at minimal power setting (10 W) [25]. This is likely because high power during RFA can lead to rapid tissue charring, which increases tissue impedance and reduces the effectiveness of the ablation [27,28]. Few RFA devices incorporate a cooling mechanism to prevent excessive charring, allowing more extensive and effective ablation zone by maintaining optimal energy flow through the tissue [29]. Slow methodological energy deposition is more effective than a sudden rise in temperature. In the study by Crino et al., lower power (30 W) applied for a longer time (50 s) resulted in a greater but slower damage [24]. Moreover, longer durations (5 min) have shown to be associated with retroperitoneal fibrosis and intestinal adhesions in an experimental study [17].
5. Immunomodulatory effect: Beyond direct thermal destruction, RFA is also believed to stimulate immunomodulation. A post-RFA increase in cytotoxic T cells and dendritic cells suggests the stimulation of the adaptive immune system responsible for “durable response” following RFA [16,20]. The increased expression of these antigen-presenting cells is also considered to enhance the antitumor response by sensitizing pancreatic cancer to systemic therapy [30,31]. Even suboptimal RFA is believed to elicit the systemic antitumor response [17].
6. Heat sink effect: Thermal ablation efficacy is limited by tissue blood flow, which acts as “heat sink”, dissipating heat and reducing ablation volume [21]. It is important to take a note of vascularity of the lesion and nearby large vessels before proceeding to RFA.

## 3. EUS-RFA Devices and Techniques of EUS-RFA

A variety of devices are available for RFA, categorized into monopolar and bipolar devices (Table 1). These devices are either needle-type electrodes, which facilitate the direct puncture of the lesion, or through-the-needle type electrodes, which are passed through a 19- or 22-G needle [32]. Each device has a specific generator to deliver tailored energy. Only EUSRA and Habib^TM^ EUS RFA electrodes are currently FDA-approved in the USA.

There are limited data on the comparison between various RFA devices’ efficacy and the area of ablation. A comparative study of animals showed no significant difference in the area of ablation between the Habib^TM^ EUS RFA probe and the EUSRA [33]. The Habib^TM^ electrode is a 1 Fr probe that can be easily passed through a 19- or 22-G needle, and it causes necrosis of 8–10 mm when used with 5–25 W power applied for 60–120 s [34,35]. The EUSRA electrode is a needle-type electrode available in 18-G and 19-G with an exposed tip of variable length (5 to 20 mm) for ablation. The exposed tip can cause necrosis of up to 8–14 mm when used with 10–50 W power applied for 10–20 s [12,36,37,38,39]. The choice of electrode, particularly the length of the exposed tip, should be tailored to the size of the tumor to optimize ablation outcomes. Theoretically, the RFA probe can induce up to a 3 cm ablation area with a single application, and larger lesions may need more applications in different parts of the tumor during the same session while keeping a 5–10 mm safety margin.

Procedural and technical aspects of EUS-RFA are described in Table 2. It is crucial to take preliminary precautions before EUS-RFA. After encountering severe adverse events (AEs) in two initial patients, including acute infective necrotizing pancreatitis and small bowel perforation, Barthet et al. recommended several prophylactic measures like: (1) the placement of a rectal nonsteroidal anti-inflammatory drug (NSAID) to reduce the pancreatitis risk, as recommended prior to endoscopic retrograde cholangiography (ERCP); (2) a prophylactic antibiotic to prevent infection; and (3) cyst fluid aspiration in cystic lesion prior to RFA to mitigate damage to an adjacent organ (from the excessive application of RFA) in the presence of a liquid component. With the change in study protocol, the authors observed a significant decrease in adverse event rate from 10% to 3.5% [40]. Additionally, considering prophylactic PD stenting is advised if the PD is within 2 mm of the lesion [38].

Post-RFA, contrast harmonic EUS (CH-EUS) offers immediate insight into the ablation zone in real time [39]. However, post-RFA hyperemia can pose a difficulty in CH-EUS interpretation, therefore, it is advisable to wait for at least a week for the evaluation of treatment response [41]. In centers with no access/ability to perform CH-EUS, contrast-enhanced CT can be performed to evaluate for treatment effect. Following the procedure, monitoring patients for symptomatic improvement and potential adverse events (AE) is essential. Clinical improvement can be observed immediately in functional PNET, whereas non-functional PNETs and other pancreatic tumors require follow-up with cross-sectional imaging to assess the radiological progress.

## 4. EUS-RFA for Pancreatic Neuroendocrine Tumor

Pancreatic neuroendocrine tumors (PNETs) are classified as functional and non-functional (NF) tumors based on their hormone secretion status [42,43]. Functional PNETs like insulinoma are typically slow-growing tumors with a lower malignancy risk [44]. Most PNETs are sporadic, small, and benign tumors, with a minority associated with inherited syndrome [multiple endocrine neoplasia (MEN)] [43]. NF PNET accounts for 70–90% of PNETs, which are increasingly identified through enhanced imaging techniques [32,45].

Current guideline from the European Neuroendocrine Tumor Society recommends surveillance for patients with asymptomatic ≤1 cm PNET and personalized management of patients with 1–2 cm PNET (without main PD dilatation) based on the type of surgical resection needed, as well as a patient’s comorbidities [2]. Active surveillance requires a patient’s compliance and involves regular cross-sectional imaging, which can significantly increase the economical, physical, and mental burden on patients. Although surgery remains the primary treatment for all types of NET, it is associated with high morbidity and various adverse events like delayed gastric emptying (5–18%), pancreatic fistula (14–58%), bleeding (1–7%), exocrine and endocrine insufficiency (27–58%), and in-hospital mortality (3–6%) [4,5]. EUS-RFA presents a minimally invasive and safer alternative to surgery for small PNET (<2 cm). Rossi et al. were pioneers in demonstrating the utility of EUS-RFA for NF PNET [46]. Subsequently, numerous studies have validated the feasibility, safety, and effectiveness of EUS-RFA for PNET (Table 3) [14,15,38,39,40,47,48,49,50,51,52,53,54,55,56,57,58]. A meta-analysis of 11 studies involving 292 patients reported the pooled technical success rate of 99.2% [95% Confidence interval (CI) 97.9–99.9%] and a complete radiological response in 87.1% (95% CI 80.1–92.8%) with EUS-RFA [12]. Therefore, while guidelines recommend surveillance for NF PNET lesions under 2 cm, there can be cases where EUS-RFA could be considered as a therapeutic option such as rapid growth or morphological changes if the tumor is growing rapidly or developing high-risk features, but surgery is deemed too risky or undesirable. Also, for high-risk patients with significant comorbidities, EUS-RFA offers a way to control tumor growth without subjecting them to the risks of surgery. In some cases, patients may prefer proactive treatment to avoid the psychological burden of long-term surveillance and the uncertainty of potential tumor growth.

The clinical efficacy of EUS-RFA for PNET ranged from 67 to 100% [15,40,47,48,49,52]. An immediate response to RFA was observed in patients with insulinoma, however, NF PNET required follow-up cross sectional imaging. Barthet et al. conducted the first multicenter prospective study in 12 patients, with 14 NF PNET undergoing EUS-RFA. A significant response was observed in 71.4% at 6 months, and 85.7% at 12 months, with a sustained response in a long-term follow-up over an average of 42.9 months. A delayed response to RFA was observed in 14–15% patients, which was attributed to the immunomodulatory properties of RFA [40,54].

In insulinomas, where hypoglycemia poses a life-threatening risk, symptomatic improvement is of particular concern. Lakhtakia et al. (2016) achieved normoglycemia within 48 h of EUS-RFA in three patients, with this response enduring for an average of 11.5 months [15]. Oleinikov et al. (2019) reported normoglycemia within an hour of EUS-RFA in seven insulinoma patients [48]. Borreli de Andreis et al. (2023) observed a sustained response in 10 patients with insulinoma over median follow-up of 19.5 months. A 19-G (EUSRA) electrode with a 5–10 mm exposed tip was used with RFA energy of 10–50 W in 1–14 cycles. A total of 90% responded to a single session, though one patient with a large tumor (19 mm) required a second session due to ongoing hypoglycemia symptoms [39].

In the largest study of EUS-RFA on the pancreatic lesion of 100 patients by Napoleon et al., encompassing 64 PNETs, a partial response to EUS RFA was noted in 31.6% and no response was noted in 9% patients [14]. Multivariate analysis revealed that presence of PNET and smaller tumor (<20 mm) significantly influenced complete tumor ablation [14]. Similarly, a systematic review by Imperatore et al. observed that larger tumor were likely to predict treatment failure (21.8 + 4.7 mm in non-responder vs. 15.07 + 7.34 mm in responder, *p* = 0.0048), whereas tumor location and type of PNET did not significantly impact the response rate [10]. Effectiveness in functional (100%) and NF PNET (93%) was comparable (*p* = 0.3). In conclusion, the overall efficacy of the EUS-RFA primarily hinges on the PNET size.

EUS-RFA for PNET is safe with a pooled incidence of AE seen in 20%, and severe incidents in <1% of cases [12]. To date, no randomized controlled trials (RCTs) have directly compared EUS-RFA with surgery for PNET. Crino et al. conducted a propensity-matched analysis by comparing EUS-RFA to surgery for insulinoma, reporting comparable efficacy (95.5% vs. 100%, *p* = 0.160), a better safety profile with minimal adverse events (18% vs. 61.8%, *p* = 0.0001) and shorter hospitalization (3.4 + 3 days vs. 11.9 + 9.7 days, *p* < 0.001) with EUS-RFA. Although, insulinoma recurrences were greater in the EUS-RFA group (16.9%) compared to surgery (0%), more than two thirds (73%) of recurrences were managed by repeat sessions of RFA [38]. High-grade tumors and the PNET part of MEN type I are at risk of being malignant, multifocal, and likely to recur, requiring pancreatic resection [59]. Therefore, EUS-RFA can be reserved for single, low-grade, sporadic PNET with the lesion size preferably < 20 mm [10,13]. Until evidence establishes it as a standard treatment, EUS-RFA remains an option for patients with comorbidities, limited life expectancy, or those preferring to avoid surgery [41,47,48,53,54].

The necessity for post-RFA surveillance in PNET is uncertain due to the tumor’s sporadic and indolent nature. Barthet et al. reported two failures with liver metastasis in one patient (grade 2 NET) following two sessions of RFA for a persistent lesion. The authors suggested surgery for RFA failure, even though a delayed response can occur in 14% at 1 year of follow-up [51]. Therefore, further RCTs are essential to evaluate EUS-RFA’s long term effectiveness, post-RFA recurrences, and overall survival compared to surgery.

## 5. EUS-RFA for Pancreatic Cystic Lesion

Pancreatic cystic lesions (PCL) are increasingly detected with an incidence of 2.4–20% in adults due to the heightened use of cross-sectional imaging [60,61]. PCL are categorized into benign lesion-like serous cystic neoplasm (SCN), lesions with malignant potential like mucinous cystic neoplasm (MCN) and intra-ductal papillary mucinous neoplasm (IPMN), and low-grade malignant lesion-like solid pseudo-papillary epithelial neoplasm (SPEN) [62]. IPMNs are further classified into main duct, branch duct, and mixed type. Branch duct IPMNs are frequently encountered in clinical practice, with a malignancy risk of 3.3–15% over 15 years [63]. PCLs exhibit complex morphology and can appear as unilocular, oligolocular, multilocular, and with or without a mural nodule/solid component. Additionally, they may or may not communicate with PD.

Current guidelines advocate surgery or surveillance for mucinous PCLs, based on the presence of high-risk stigmata or worrisome features [3]. Studies have validated the efficacy of EUS-guided ablation for unilocular or oligolocular (<6 locule) PCL with malignant potential, or progressively enlarging cysts (2–6 cm) in patients with good life expectancy or high surgical risk [64,65]. Although EUS-guided ethanol ablation has been widely utilized, its application in PCL is limited by significant AE (10–16%) [61,64,66]. There is growing interest in EUS-RFA for PCL due to its safety profile (Table 4). Pai et al. were the first to report the feasibility and safety of EUS-RFA in six patients with PCL in the head region (MCN: 4, IPMN: 1 and mucinous cystic adenoma: 1) [34]. The Habib^TM^ probe was used for EUS-RFA. The complete resolution of cysts was observed in 33.3% patients, with a partial response in 50% at a mean follow-up of six months. No major complications were reported, with only mild abdominal pain in two patients [34].

The efficacy of EUS-RFA in PCL with high risk stigmata, including the presence of a mural nodule, has been assessed, demonstrating significant diameter reduction in PCL size with complete nodule disappearance [14,40,51,52]. In a multicenter prospective study by Barthet et al. involving 29 patients, 16 with IPMN and mural nodules (>5 mm) in 12 patients, a significant response was observed in 65% at 6 months and 71% at 12 months, with the disappearance of all mural nodules (100%) [40]. The long-term follow-up of these patients over three years showed a similar response rate of 66.6%, with failure in 33.3% patients. Further analysis indicated that failures had larger cysts (median 35 mm, range 25–76 mm) compared to the responders (median size 12 mm, range 9–32 mm). Moreover, two patients developed adenocarcinoma in the tail region after 26 and 42 months in patients with IPMNs in the head region with size > 40 mm [51]. Younis et al. observed a complete response in 60%, and partial in 20%, of 5 PCL (IPMN: 4, MCN: 1), with a mural nodule in 3 IPMN. In one patient with failure after a second session of RFA, malignancy was detected on the FNA of the mural nodule [52]. Napoleon et al. observed a similar response rate in their largest study on pancreatic lesion, with a complete response in 62.5% patients and a partial response in 32.7% among 10 IPMN with mural nodules in 12 (size 5–30 mm) [14]. Choi et al. evaluated EUS-RFA in two patients with SPEN, reporting a complete radiological response in one patient (50%) with no response in others (50%) [47]. These findings highlight the potential of EUS-RFA in managing PCLs with diverse outcomes.

In addition to studies on PCL with malignant potential, Oh et al. explored the safety and efficacy of EUS-RFA in 13 patients with symptomatic SCN [67]. SCN are benign and usually asymptomatic tumors with a microcystic component [68]. Ablating SCN can be challenging due to their multi-septated nature. In this study, seven patients underwent a single session, and six received a second session. Only a partial response was observed in 61.5% patients at median follow-up of 9.2 months with no complete response [67]. The lower response was attributed to the complex cyst structure, including honeycombing and multiple septation, which impeded complete ablation.

Collectively, these studies confirm the technical feasibility and safety of EUS-RFA for PCL, with a varying response rate from 33 to 71%, and fewer AEs [14,34,40,51,52]. The variation in response rate may result from the differing definition of response across studies. Despite a promising response rate, post-RFA surveillance is crucial for IPMN (especially > 40 mm), as RFA of IPMN unlikely eliminates the risk of developing concomitant pancreatic malignancy [51]. Long-term follow-up is necessary to monitor the risk of concomitant malignancy, which has been reported in 4–8.8% patients over 3–5 years [69]. Given the need for post-RFA surveillance in IPMN patients to detect concomitant malignancy, the justification for performing EUS-RFA warrants consideration.

## 6. EUS-RFA for Pancreatic Cancer

Pancreatic cancer is the third leading cause of cancer related deaths in the USA, and the seventh worldwide [70]. It is an aggressive malignancy and often detected at an advanced stage. Although surgery is the mainstay of treatment, approximately 80% of sufferers are ineligible due to advanced disease at the time of diagnosis [31]. Despite advances in chemotherapy, radiotherapy, and the surgical resection technique, the overall five-year survival rate has not improved and remains less than 20% [22,71]. While the majority of patients experience disease progression, newer chemotherapeutic drugs can help in down-staging the tumor in 25–30% of cases [72]. Local RFA is explored as an adjunct to chemotherapy for down-sizing and down-staging locally advanced and unresectable pancreatic cancer. However, the application of percutaneous and intra-operative RFA is limited by factors like tumor accessibility and a high AE rate [73,74]. EUS overcomes these limitations with real-time imaging and precise targeting. Several studies have shown the safety of EUS-RFA in advanced and metastatic pancreatic cancer (Table 5) [17,18,19,22,24,75,76,77,78]. Arcidiacono et al. (2012) conducted the first feasibility study on 22 patients with locally advanced pancreatic cancer using a 14-G bipolar cryotherm probe [75]. All patients received gemcitabine-based chemotherapy and six received chemo-radiation therapy. Despite a 72.8% technical success rate, the study reported high AEs in 36.4% and failed to demonstrate impact on tumor size. Additionally, difficulty in inserting the cryotherm probe in six patients was observed due to a stiff gastrointestinal wall and tumor.

Song et al. in 2016, shared their initial experience with an 18-G EUSRA electrode in six patients with unresectable pancreatic cancer (stage III: 4 and stage IV: 2). Pancreatic cancer was located in the head in four, and in the body in two patients, with an average size of 3.8 cm (range 3–9 cm). Power of 20–50 W was applied for 10 s, and repeated until the hyperechoic zone sufficiently covered the tumor. This approach achieved 100% technical success, with only two patients experiencing mild abdominal pain [17]. Post-EUS-RFA, CH-EUS indicated a non-enhancing ablated area with an increased blood flow in the surrounding zone, suggesting the potential enhancement of chemotherapy effectiveness. Therefore, gemcitabine was administered to three patients on the same day. However, this was a feasibility study with only four-months follow-up with inconclusive evidence of EUS-RFA effectiveness [17]. High technical success with 18-G EUSRA can be attributed to its flexibility and maneuverability compared to the 14-G cryotherm probe.

Further studies have reported varied responses to EUS-RFA in pancreatic cancer, observing partial tumor reduction [18,19,24,76]. Wang et al. noted an increase in apparent diffusion coefficient on MRI and 20% tumor ablation, which was associated with improved overall survival (1 year) in one patient who received multiple rounds of ablation [19]. Similarly, Crino et al. reported average tumor ablation of 30% (5.8–73.5%) in seven locally advanced pancreatic cancer patients and one metastasis from renal cell carcinoma (RCC) using lower power, i.e., 30 W for an average of 50 s or more and slow thermal damage. This approach resulted in 100% technical success with mild abdominal pain observed in only three patients [24].

For pancreatic cancer, the immunomodulating property of RFA is thought to bolster the systemic immune response against the tumor, potentially enhancing the chemotherapy efficacy and improving overall survival [16,31]. Oh et al. provided long-term outcomes in 22 patients with unresectable pancreatic cancer who received both EUS-RFA and sequential gemcitabine based chemotherapy. Twenty patients (95.5%) experienced treatment failure. However, the median overall survival was 24.03 months, with a median progression free survival of 16.37 months. On univariate analysis, tumor extent (locally advanced vs. metastatic) and the time interval from diagnosis to EUS-RFA was associated with overall survival and progression free survival, respectively [77]. Similarly, Thosani et al. reported median survival of 20.5 months (95% CI, 9.93–42.2 months) in 10 patients, out of which two patients survived beyond 61 and 81 months follow-up following EUS-RFA. Tumor regression was observed in 60% with significant downsizing enabling pancreaticoduodenectomy in 1 patient. All the patients in this study received concurrent chemotherapy. A significant decrease in CA 19–9 level was observed, however, the worsening of existing abdominal pain was noted in 55% of patients [22]. As this was a single cohort study, treatment effect might have been biased favorably towards the RFA. A prospective open label study by Kongkam et al. compared chemotherapy plus EUS-RFA (n = 14) vs. chemotherapy (n = 14). Significant necrosis of the tumor (combination: 100% vs. chemotherapy alone: 50%, *p* = 0.014) was noted, with a reduced narcotic requirement from 63.6 to 37.1 mg in the combination group, while no change in narcotic dosage was seen in the chemotherapy alone group. However, there was no difference in mortality at six months of follow-up [78]. This preliminary non-randomized study highlights the potential benefits of combining EUS-RFA with chemotherapy, but underscores the need for larger randomized controlled trials (RCTs) with longer follow-up to confirm these findings, alongside the standardization of RFA techniques.

## 7. EUS-RFA for Other Indication

Pancreatic metastases are rare, primarily from the RCC. Metastasis from RCC is often hypervascular, associated with long-term survival [79]. Focal therapies have become standard treatment for oligometastatic RCC. EUS-RFA is recognized as safe and effective for treating pancreatic metastasis from RCC. In the prospective series by Chanez et al., 12 patients (median age 70.5) with 21 pancreatic metastases from RCC underwent 26 EUS-RFA procedures. For seven patients, RFA was the only treatment. The focal control rates at 6 and 12 months were 84% and 73%, respectively. Major AEs occurred in two patients only—one developed a para-duodenal abscess while on concurrent tyrosine kinase inhibitor, and another with a pre-existing biliary stent developed a hepatic abscess [80]. Conversely, Ferreira et al. reported a lower EUS-RFA response rate in RCC metastasis, with only one out of nine patients showing a response [56]. Similarly, Napoleon et al. noted a less favorable response in pancreatic metastasis (complete response in 30%, partial in 43.5% and no response in 26%) compared to the other pancreatic lesions. Of 23 patients with pancreatic metastasis, 19 had RCC [14]. There is no information on concurrent chemotherapy in both studies. Given these contradictory results, further research is required to clarify the role of EUS-RFA in pancreatic metastasis.

EUS-RFA for celiac ganglia has been shown to improve the pain score [81]. Bang et al. conducted an RCT comparing EUS-RFA for celiac ganglia (n = 12) with EUS-guided celiac plexus neurolysis (CPN) (n = 14). EUS-RFA provided more pain relief and improved quality of life compared to EUS-CPN [82]. Jiang et al. performed EUS-CPN before the EUS-RFA of a pancreatic lesion in seven patients. Significant relief in abdominal pain was observed in six patients, suggesting that EUS-CPN may be more effective when combined with EUS-RFA [76].

The efficacy of EUS-RFA for lymph nodes has been reported in animal studies and case reports [35,83]. However, its advantages for treating isolated lymph node metastasis in a pancreatic tumor warrants further investigation.

## 8. Safety of EUS RFA

EUS-RFA is associated with a spectrum of mild to severe AEs, with an incidence of 14–22% [6,10,12,13,14]. These AEs include abdominal pain, gastric and intestinal wall burns, gastric wall hematoma, pancreatitis, bleeding, splenic hematoma, peritonitis, bowel perforation, PD stenosis, duodenal stricture, diabetes, and abscess [14,17,18,24,34,38,39,40,47,48,49,50,52,53,54,55,58,75,80]. The majority of AEs are mild–moderate grade, with severe AE occurring in 0.5–0.9% [10,12,13]. The most common AE of EUS-RFA is mild abdominal pain followed by pancreatitis, with a reported incidence of pancreatitis in 7.9–10% [12,14,38,47,78]. The proximity of the lesion to the main PD significantly influences the risk of post-RFA pancreatitis, underscoring the importance of preventive measures such as prophylactic rectal NSAIDs placement and PD stenting for lesions close to the PD. Napoleon et al. in their study of 100 patients reported that the close proximity (≤1 mm) to the PD was an independent risk factor for AEs [14]. Similarly, Crino et al. found that 89% of patients with pancreatitis had PD located <2 mm from the lesion [38]. Although there are limited data on the role of PD stenting, and no consensus on the required distance from the lesion, a minimum 2 mm of distance can be considered for prophylactic PD stenting. ERCP PD stenting can also cause pancreatitis, therefore it is advisable to keep a minimum gap of three days to observe for post-ERCP events [67]. Considering limited data on PD stenting, further studies with large sample sizes are needed to clarify its role in preventing post-RFA pancreatitis.

Most AEs can be managed with conservative treatment, and only 3–16% of patients can require additional intervention in the form of EUS-guided cystogastrostomy, ERCP-stenting, or surgery [14,40,53]. Bleeding following RFA is infrequent, typically occurring from the puncture site, and can be effectively managed with endoscopic techniques, such as an adrenaline injection or clipping [53,55,58,75].

**Figure 2:**
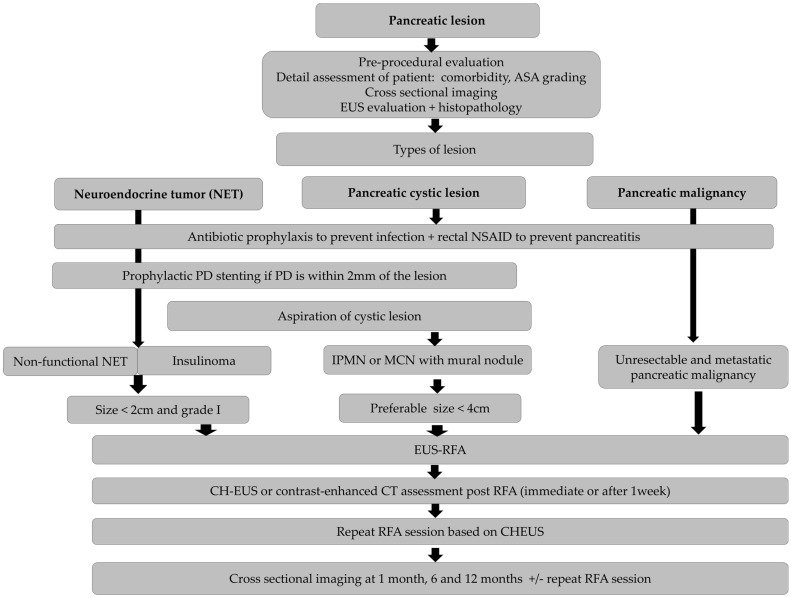
Proposed algorithm for EUS RFA of pancreatic neoplasia.

Although EUS-RFA is considered safe, one death has been reported due to the development of infected retrogastric collection in a 97-year old with multiple comorbidities [54]. Overall, while EUS-RFA is safe, it necessitates careful patient selection and preventive strategies to mitigate the risk of AEs (Figure 2).

## 9. Conclusions and Future Perspectives

EUS-RFA is a promising, minimally invasive ablative technique for pancreatic lesions, offering a safer alternative for benign, premalignant, and certain malignant conditions. Its efficacy in treating PNET and cystic lesions underscores its potential. However, the absence of RCTs comparing EUS-RFA to surgical methods highlights a significant gap in the literature, with most studies being observational with small sample sizes and limited follow-up. There is an RCT underway evaluating the efficacy of EUS-RFA compared to surgery for insulinomas (ERASIN-RCT as registered on clinicaltrials.gov), and also other prospective studies evaluating outcomes of EUS-RFA in PDAC, along with the standard of care in neoadjuvant chemotherapy (PANCARDINAL-1 trial as registered on clinicaltrials.gov).

Future direction should focus on standardizing procedural parameters such as electrode types, power setting, and ablation duration to optimize outcomes and facilitate comparison across studies.

Particular attention is needed on the management of PCLs with EUS-RFA, especially regarding the necessity and frequency of post-ablation surveillance. Well-designed RCTs with extensive follow-ups are essential for understanding EUS-RFA’s long-term safety, efficacy, and impact on survival rates.

Investigating EUS-RFA’s role alongside systemic therapies could reveal new combination treatment avenues, especially for pancreatic adenocarcinoma.

In conclusion, EUS-RFA holds considerable promise as a treatment modality for pancreatic lesions, offering a blend of minimal invasiveness and therapeutic potential. As the technique continues to evolve, ongoing research and clinical trials will be crucial in defining its role, refining procedural practices, and ultimately enhancing patient care in pancreatic disease management.

## Figures and Tables

**Table 1:** Devices for pancreatic RFA.

	Type of Electrode	Tip Size	Length of Active Segment (mm)	Length of the Catheter	Internal Cooing	Manufacturer	City	State
**Monopolar RFA**								
HabibTM EUS-RFA	Through the needle	1 Fr	20	220 cm	No	EMcision Ltd.	London	UK
19-G EUS-RFA	Needle type	19 G	10–15	-	No	Radionics, Inc.	Burlington	Massachusetts (USA)
EUSRA	Needle type	18 G, 19 G	5, 7, 10, 15, 20	150	Yes	STARmed	Koyang	South Korea
**Bipolar RFA**								
Hybrid-cryotherm probe	Needle type	14 G	26	-	Yes	ERBE	Tubingen	Germany

**Table 2:** Procedural aspect of pancreatic RFA.

**Preprocedural parameters** Indication of RFA. Assess for lesion with diagnostic EUS: size, site, resectability, and vicinity to the pancreatic duct or major vessels. Endoscopic retrograde cholangiopancreatography and pancreatic duct stenting is suggested if the lesion is within 2 mm of the PD. Withhold anti-thrombotic and avoid RFA if PT INR > 1.5 and platelets < 50,000 per microliter. Patient is kept fasted for >6 h before the procedure. Informed consent is obtained from the patient.
**Accessories required for the procedure** Linear or forward view therapeutic EUS scope. RFA needle type electrode or 19/22-G FNA needle for through the needle electrode. Radiofrequency (RF) generator: to deliver the energy. Peristaltic pump (Included with STARmed device) for cooling the RFA needle during ablation by circulating normal saline. Connecting cables to connect RFA needle to the RF generator. Connecting tubes (blue and white tubes) to connect RFA needle to the peristaltic pump. Ground pads (two).
**System set-up required for EUSRA** Turn on the RF generator (VIVA, STARMED, Koyang, Republic of Korea) by pressing the power (start/stop) button. Place ground pads on patient thighs and connect the pads cable to VIVA RF generator. Connect the needle cooling tubes to the peristaltic pump containing refrigerated saline solution (32 °F), and to the needle. Connect electrode cable to RF generator and the RFA needle. Select “Continuance mode” on the generator by pressing the mode button until display window shows the “Continuance mode”.
**Technical aspects** Procedure is performed under monitored anesthesia care and prophylactic antibiotics. It is suggested to apply rectal non-steroidal anti-inflammatory drugs (NSAID) prior to the procedure. Patient is positioned in left lateral decubitus position. Exposed tip length of RFA electrode needle is chosen based on the lesion size. Once the lesion is visualized, the lesion is brought in the center, and vessel and duct-free area are chosen by using color Doppler. The distal (difficult-to-reach) area is targeted first. Once the tip of the electrode is visualized within the lesion, the RF generator is activated to deliver 10 to 50 W ablation power in 1–5 cycles with each cycle of 10–20 s. During ablation hyperechogenic bubbles start appearing. Stop RFA if hyperechoic bubble appears or the impedance exceeds ≥ 500 Ω. It is suggested to start from the right most distal part of lesion first and then gradually withdraw the needle and cover all the areas by additional passes with fanning method. For smaller lesions (<1 cm), the needle can be kept in the center for ablation. A 5 mm safety margin from the surrounding structure is maintained. Real-time evaluation of the ablation zone can be performed with contrast harmonic EUS (CH-EUS) post-RFA. However, it is advisable to wait for at least 1 week for assessment as post-RFA hyperemia can influence the reading if done immediately.
**Post-procedure** Post procedure patient is hospitalized. Blood sugar levels are monitored regularly during and post procedure in patients with Insulinoma. After discharge, patients are followed up at 1 month, 4–6 months and yearly thereafter. EUS with contrast harmonic and or cross-sectional imaging is performed on follow-up to decide regarding further ablation sessions

**Table 3:** EUS-RFA for pancreatic neuroendocrine tumor with cases ≥ 3.

	Study Design	No. of Patients with NET/No. of Lesions	Age (Mean in Years)	Size of NET Mean in mm, (Range)	Location	Type of PNETs	Reasons for RFA	RFA Device	RFA Power (W)	RFA Sessions No. of Sessions: Per Patient	Efficacy (%)	No. of Adverse Events	Follow-Up in Months, Mean
Lakhtakia et al., 2016 [15]	Retrospective	3/3	45	17 (14–22)	Head: 1 (multiple) Geu: 1 Body: 1	Insulinoma	High surgical risk: 3	19 G EUSRA	50	1:1	100	0	11.5
Choi et al., 2018 [47]	Prospective	8/8	55	19.3 (8–28)	Head: 3 Body: 5	Insulinoma: 1 NF: 7	High surgical risk: 2 Refused surgery: 6	19 G EUSRA	50	14 1:4 2:2 3:2	75	2 abdominal pain: 1 pancreatitis: 1	15.5
Barthet et al., 2019 [40]	Prospective	12/14	59.9	13.1 (10–20)	Head: 3 Body: 6 Tail: 5	NF: 14	NR	18 G EUSRA	50	1:1	86	3 pancreatitis: 1, small bowel perforation: 1, PD stenosis: 1	12
Oleinikov et al., 2019 [48]	Retrospective	18/27	60.4	14.2 (4.5–29)	Head: 10 Body: 8 Uncinate: 5 Tail: 2 Liver metastasis: 1 LN metastasis: 1	Insulinoma: 7 NF: 11 (Grade 3:2)	High surgical risk: 3 refused surgery: 13 Patient preference: 12	19 G EUSRA	50	1:1	94.5	2 (mild AP)	8.7
De Nucci et al., 2020 [49]	Prospective	10/11	78.6	14.5 (9–20)	Head: 3 Body: 5 Tail: 3	Insulinoma: 5 NF NET: 6	High surgical risk: 7 Refused surgery: 3	19 G EUSRA	20	1:1	100	2 (abdominal pain)	12
Furnica et al., 2020 [50]	Retrospective	4/4	62.7	12 (6.5–22)	Head: 2 Neck: 1 Tail:1	Insulinoma	NA	19 G EUSRA	50	1:1	100	2	22
Barthet et al., 2021 [51] *	Prospective	12/14	59.9	13.1(10–20)	Head: 3 Body: 6 Tail: 5	NF: 14	NA	18 G EUSRA	50	1:11 2:1	85.7	2 Liver metastasis: 1 Failure: 1	45.6
Younis et al., 2022 [52]	Prospective	7/7	67.4	10.7 (6–18)	Head: 2 Body: 4 Tail-1	NF: 6 (1 with liver mets) Insulinoma: 1	NA	19 G EUSRA	50	1:1	Complete response: 66.7 No response: 33.3	3 Abdominal pain: 2 Pancrearitis: 1	12
Marx et al., 2022 [53]	Retrospective	27/27	65	14 (7–25)	Head: 8 Body: 3 Tail: 11	NF: 27	Patient preference: 19 High risk: 7 Physician preference: 1	19 G EUSRA	50 in 26 30 in1	1:25 2:2	Complete response: 93	7 ** PFC in 3: Bleeding: 2 PD and CBD stricture: 1	15.7
Marx et al., 2022 [54]	Retrospective	7/7	66	13.3 (8–20)	Head: 1 Neck: 3 Body: 2	Insulinoma: 7	Patient preference: 4 High risk surgery: 3	19 G EUSRA	50	1:1	85.7	4 (Death due to infected retrogastric collection: 1)	20.7
Crino et al., 2023 [38]	Retrospective	89/89	55.1	13.4	Head: 34 Body: 39 Tail: 16	Insulinoma: 89	NA	18/19 G EUSRA	10–50	1:74 2:11	100 (technical success) Recurrence: 16.9% (73% treated by repeat RFA)	16	23
Rosi et al., 2022 [55]	Retrospective	3/3	82.7	12 (9–14)	Head: 1 Tail: 2	Insulinoma: 3	High risk surgery: 3	19 G EUSRA	30	1 in 2 patients 2 in 1 patient	100	1 (intraprocedural bleeding-clip + adrenaline)	24
Ferreira et al., 2022 [56]	Prospective	29/23	59 ^$^	NA	NR	NF: 10 Insulinoma: 13	NA	19 G EUSRA	50	NA	Insulinoma response: 100 Significant response: 73.3	14 Mild pain: 4 Mild AP: 3 Others: 5 ^$$^	9.5
Borrelli de Andreis et al., 2023 [39]	Retrospective	10/10	67.1	11.9	Head: 3 Tail: 7	Insulinoma	High risk surgery: 7 Patient preference: 3	19 G EUSRA	20–50	1:9 2:1	100	2 (abdominal pain)	19.5
Napoleon et al., 2023 [14]	Retrospective	100 ^#^/64	NR	15	NA	NF: 48 Function: 16	NA	19 G EUSRA	50		Complete-60.2 Partial-31.6	22 ^##^ (2 EUS cyst drain, 1 PD and CBD stenting) 3 CP	12
Debraine et al., 2023 [57]	Retrospective	11/11	65	11.6	Head: 5 Neck: 1 Body: 3 Tail: 2	Insulinoma	High risk surgery: 6 Patient preference: 5	EUSRA	50	1:10 2:1	100 Radiological response: 63.6	4 (abdominal pain)	26
Biermann et al., 2024 [58]	Prospective	3/3	67.33	13.33	Tail: 1 Uncinate: 1 Head: 1	Insulinoma	High risk surery: 3	19 G EUSRA	20	1:1	100	1 (mild Hematochezia)	12.66

Abbreviations: LN: lymphnode, NF: nonfunctioning, PNET: pancreatic neuroendocrine tumor, RFA: radiofrequency ablation. *: This is a long-term follow-up of study published in 2019 by Barthet et al. and colleagues [40]. **: out of 7, patients 3 had PFC. Endoscopic cystogastrostomy was performed on all 3, however, due to persistent fistula even after cystogastrostomy, one required surgery. Bleeding was seen in 2 patients—one was managed with adrenaline injection and another required clipping. One patient with PD and CBD stricture underwent stenting for both. ^$^: Included 29 patients—10 with NF PNET, 13 with insulinoma, 1 adenocarcinoma, and 11 with metastasis. Median age of all 29 patients. Separate age of patients with NET not provided. ^$$^: Separate AEs for NET are not provided. Five other events include: one minor bleed, one case of main pancreatic duct stenosis, one case of post-procedural fever, one case of gastric wall hematoma, and one case of post-anesthesia urinary retention. ^#^: In this study, 100 patients had 104 lesions, out of them 64 were NET. ^##^: 22 patients out of 100 developed adverse events. Adverse events rate in NETs was not specified separately.

**Table 4:** EUS RFA in pancreatic cystic neoplasm (PCL).

Author	Study Design	No. of Patients with PCL/No. of Lesions	Age (Mean in Years)	Size of PCL Mean in mm (Range)	Location	Type of PCL	RFA Device	RFA Power (Watt)	RFA Sessions	Efficacy (%)	No. of Adverse Events	Follow-Up in Months
Pai et al., 2015 [34]	Prospective	6/6	60	36.5 (24–70)	Head: 6	MCN: 4 IPMN: 1 MCA: 1	Habib^TM^ passed through 19/22 G	5–25	1:1	CR: 33. PR: 50	2 mild abdominal pain	6
Choi et al., 2018 [47]	Prospective	2/2	37	21.5 (20–23)	Head: 1 Tail: 1	SPEN: 2	19 G EUSRA	50	1:1	CR: 50 PR: 50	0	15.49
Barthet et al., 2019 [40]	Prospective	17/17	65.7	28 (9–60)	Head: 10 Body: 4 Tail: 3	IPMN: 16 MCN: 1	18 G EUSRA	50	NA	CR: 71 Failure: 29	1 (small bowel perforation)	12
Oh et al., 2021 [67]	Retrospective	13/13	60	50 (34–53)	Head/Uncinate: 5 Body/tail: 8	SCN: 13	19 G EUSRA	50	7:1 6:2	CR: 0 PR: 62 NR: 38	1	9.2
Barthet et al., 2021 [51] ^#^	Prospective	17/17	65.7	29.1	Head: 10 Body: 4 Tail: 3	IPMN: 16 MCN: 1 MN: 12	18 G EUSRA	50	NR	CR: 66 Failure: 33.3	2 distant pancreatic adenoca	45.6
Younis et al., 2023 [52]	Prospective	5/5	77.6	36 (12–60)	Head: 3 Body: 2	IPMN: 4 MCN: 1 MN: 3	19 G EUSRA	50	1:3 3:2	CR: 50 PR: 20 Failure: 20 (malignancy)	3 (1 AP, 2 mild pain)	7
Napolean et al., 2023 [14]	Retrospective	100/11	NR	29	NR	IPMN: 10 SPEN: 1 MN: 10	19 G EUSRA	50	NR	CR: 62.5 PR: 37.5	NR	13 months

Abbreviations: CR: complete response, IPMN: intraductal papillary mucinous neoplasm; MCA: microcystic adenoma; MCN: mucinous cystic neoplasm; MN: mural nodule, NR: not reported, PR: partial response, RFA: radiofrequency ablation. ^#^: This is a long-term follow-up of the study published in the Endoscopy journal in 2019 by Barthet et al. [40].

**Table 5:** EUS RFA in pancreatic cancer (PC).

Author	Study Design	No. of Patients/No. of Lesions	Age (Mean in Years)	Size of Tumor Mean in mm (Range)	Location	Type of Lesions	RFA Device	Technical Success (%)	Outcomes	No. of Adverse Events	Overall Survival Months, Means
Arcidiacono et al., 2012 [75]	Prospective	22	61.9	35.7(23–54)	Head and Neck: 16 Body: 4 UP: 2	LAPC	HybridTherm probe	72.7	-	8 (36.4%) Early: Abdominal pain: 3 Duodenal bleeding: 1 -Clipping Late Jaundice: 2 stenting Duodenal stricture: 1 Cystic collection: 1	5.6
Song et al., 2016 [17]	Prospective	6	62	48 (30–90)	Head: 4 Body: 2	Stage 3: 4 Stage 4: 2	18 G EUSRA	100		Mild abdominal pain: 2 (33%)	NR
Scopelliti et al., 2018 [18]	Prospective	10	45	49.2 (25–75)	Head: 4 Body: 6	unresectable	18 G EUSRA	100	Decrease in tumor size (50%); stable disease (50%)	Mild abdominal pain: 2 (20%) ascites: 2 (20%) Peri-pancreatic fluid: 2 (20%)	NR
Crino et al., 2018 [24]	Retrospective	9 (excluded 1 with necrotic component)	67.5	36 (22–67)	Head: 3 Body: 2 UP: 2	LAPC:7 Renal Metastasis:1	18 G EUSRA	100	mean tumor ablation 30%	Mild abdominal pain: 3 (33%)	NR
Jiang et al., 2021 [76]	Prospective	8	74.3	46.9 (38–58)	Head: 5 Body: 2 Tail: 1	LAPC: 8	Habib™	100	Significant reduction in tumor size at 1 month (mass reduced by 34.3%)	Mild abdominal pain 1 912.5)	10.7
Wang et al., 2021 [19]	Retrospective	11	64.7	28 (17.2–38)	Head: 4 Neck: 3 Body: 3 Tai: 1	Stage 3: 7 Stage 4: 4	Habib™	100	Tumor size decreased in 2; CA19–9 decreased in 5 patients	Mild abdominal pain: 2 (18%)	5.2
Oh et al., 2022 [77]	Prospective	22	60.5	38 (32.8–45)	Head: 14 Body: 4 Tail: 3 On resected margin:1	Stage 3: 14 Stage 4: 8	19 G EUSRA		Treatment failure:95.5%	Abdominal pain: 3 (13.6%) Peritonitis:1 (4.5%)	24
Thosani et al., 2022 [22]	Prospective	10	62.3	39.2 (14–68)	Head: 4 Neck: 2 Body: 2 Tail: 2	Stage 3: 7 Stage 4: 3	Habib	100	Tumor regression: 6 (60%) Tumor progression:2 (20%) Decrease in Ca19.9 levels	Worsening of abdominal pain: 12 (55%)	20.5
Kongkam et al., 2023 [78]	Prospective	14	66.3	59.7 (49.8–66.5)	NR	Stage 2: 1 Stage 3: 3 Stage 4: 10	19 G EUSRA	100	Tumor necrosis: 100% Reduced narcotic use from 63.6 to 37.1 mg	Mild pancreatitis in 1 (7.3%)	6
